# Mesothelial/Monocytic Incidental Cardiac Excrescences (MICE): An Enigmatic Cardiac Pathology

**DOI:** 10.7759/cureus.90788

**Published:** 2025-08-23

**Authors:** Arifa B Laskar, Biswajit Dey

**Affiliations:** 1 Pathology, North Eastern Indira Gandhi Regional Institute of Health and Medical Sciences, Shillong, IND

**Keywords:** differential histopathological diagnosis, histiocytes, mesothelial/monocytic incidental cardiac excrescences (mice), mitral valve stenosis, tumor-like lesion

## Abstract

Mesothelial/monocytic incidental cardiac excrescences (MICE) are rare, reactive tumor-like lesions of the heart. They are typically incidental findings during cardiac surgery. They can mimic thrombi or neoplasms such as myxoma, papillary fibroelastoma, and metastatic carcinoma histopathologically, leading to diagnostic confusion. We report a case of atrial MICE in a 40-year-old woman with rheumatic mitral stenosis and long-standing atrial fibrillation, who underwent mitral valve replacement following a failed percutaneous transvenous mitral commissurotomy. Histopathological examination revealed characteristic features of MICE in the left atrial appendage, with clusters of mesothelial cells and histiocytes embedded in fibrin, confirmed by immunohistochemistry. This case underscores the need for heightened awareness among pathologists to avoid misdiagnosis.

## Introduction

Mesothelial/monocytic incidental cardiac excrescences (MICE) are rare, reactive tumor-like lesions of the heart [1]. The median age of occurrence is 61.5 years, with cases reported in individuals aged five to 80 years [1]. They are composed of mesothelial, histiocytic, and varying numbers of inflammatory cells, all embedded within a fibrinous network [1]. Often discovered incidentally during cardiac surgery, these lesions can lead to misdiagnosis on histopathology. Although non-neoplastic, they can histologically mimic thrombi or tumors, posing diagnostic challenges [1-3].

It has also been suggested that MICE are part of the broader group known as "histiocytosis with raisinoid nuclei," which encompasses benign histiocytic proliferations at various anatomical sites, sharing similar histopathological characteristics [2]. The characteristic cells described in these conditions are polygonal to ovoid histiocytes with eosinophilic cytoplasm and oval to irregular or shrunken nuclei, often showing subtle grooves and inconspicuous nucleoli [2]. The most common sites include the cardiac chambers and valves, particularly the aortic and mitral valves, or freely floating within the pericardial cavity, and may be associated with prior cardiac interventions [3]. We present a rare case of atrial MICE incidentally diagnosed in a patient of rheumatic heart disease with mitral stenosis and a history of percutaneous transvenous mitral commissurotomy (PTMC), emphasizing the importance of recognizing this entity to avoid misdiagnosis and overtreatment.

## Case presentation

A 40-year-old woman presented with progressive shortness of breath and fatigue over the past six months. Initially, she experienced dyspnea on exertion (New York Heart Association (NYHA) class II), which has worsened to dyspnea at rest (NYHA class IV) in the last two weeks. The patient was diagnosed with rheumatic heart disease during adolescence, following an episode of acute rheumatic fever. She was later diagnosed with mitral stenosis at the age of 26 years after evaluation for exertional breathlessness and was started on secondary prophylaxis with benzathine penicillin. She remained relatively stable for many years under irregular follow-up.

She underwent PTMC at the age of 34 years. However, the procedure was suboptimal, with incomplete commissural separation, and symptoms persisted with only partial improvement. There was no history of coronary artery disease, diabetes, or hypertension. Follow-up echocardiograms showed residual severe mitral stenosis and progressive left atrial enlargement. Over the years, she developed atrial fibrillation and has been on rate control therapy and oral anticoagulation since.

On examination, she had mild pallor, raised jugular venous pressure (JVP), and bilateral pitting pedal edema. She had a normal body temperature, a pulse rate of 110 beats per minute, a blood pressure of 100/60 mmHg, a respiratory rate of 22 breaths per minute, and an oxygen saturation of 90% while breathing ambient air. 

With a history of a failed PTMC and now presenting with advanced symptomatic mitral stenosis and decompensated heart failure, a surgical mitral valve replacement was planned. She underwent mitral valve replacement surgery, and the excised mitral valve tissue, along with the left atrial appendage, was submitted for histopathological evaluation.

**Figure 1:**
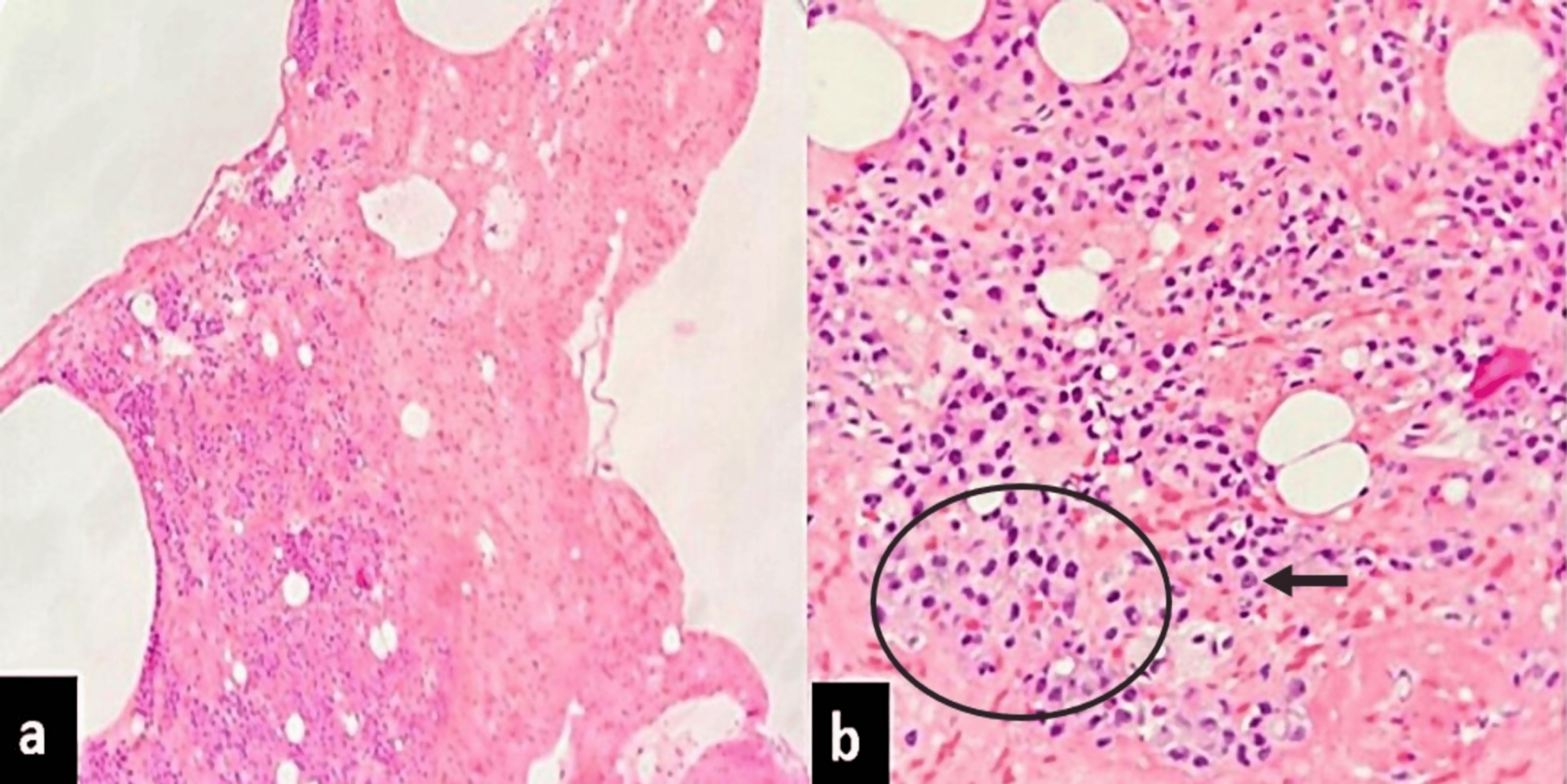
Histopathological images a: Low-power view of the lesion entrapped in fibrinous meshwork (hematoxylin and eosin (H&E), 40x); b: A cluster of polyhedral-shaped mesothelial cells (encircled) and histiocytes (arrow) is seen enclosed within fibrin (H&E, 400x)

On microscopic examination, the valvular tissue showed degeneration, and the left atrial appendage demonstrated myocyte hypertrophy. Additionally, histological evaluation revealed an incidental finding consisting of a cluster of polyhedral cells and histiocytes intermixed with fibrin and erythrocytes and focally organized into strips or tubular formations (Figures 1a-1b). There was an organization of fibrin with ingrowth of fibroblasts and early collagen deposition. However, no well-formed fibrosis or calcification was noted within the lesion.

**Figure 2:**
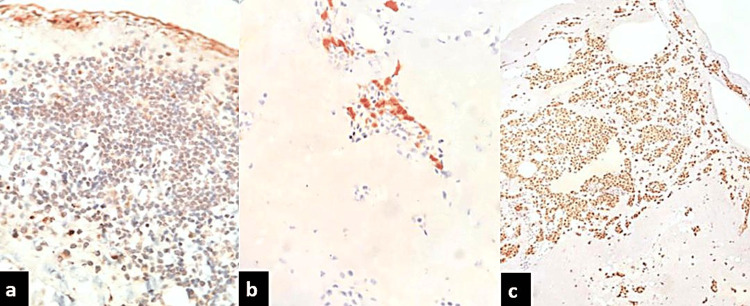
Immunohistochemical analysis a: Pancytokeratin highlighting the flat mesothelial-like layer and scattered lesional cells (immunohistochemistry (IHC), 400x); b: Calretinin highlighting the mesothelial cells (IHC, 400x); c: CD68 positivity in solid clusters confirming histiocytic differentiation (IHC, 200x)

Immunohistochemical analysis revealed positivity for calretinin, Wilms tumor protein-1 (WT1), and pancytokeratin in the mesothelial cells, while histiocytes showed strong CD68 staining (Figures 2a-2c). Epithelial membrane antigen (EMA) and CD31 were negative. A diagnosis of mesothelial/monocytic incidental cardiac excrescence was made. The patient was discharged after one week of surgery and was advised to undergo regular follow-up.

## Discussion

Mesothelial/monocytic incidental cardiac excrescences is a rare, benign proliferative cardiac lesion that was first identified by Rosai et al., who initially referred to it as histiocytoid hemangioma [4]. The term 'MICE' was later proposed and formally established by Veinot et al. in 1994 [5]. As of 2024, approximately 60 cases of MICE have been reported in the English-language medical literature. This number reflects a gradual increase from earlier counts, with 43 cases noted in 2014 and around 50 cases by 2018 [1,6]. The rise in reported cases may be attributed to heightened awareness and improved histopathological recognition of this rare entity.

The etiology of cardiac MICE remains unclear. Two main hypotheses have been proposed to explain their origin: the 'reactive' theory, which suggests a response to injury or inflammation, and the 'artifact' theory, which considers them a result of procedural or handling artifacts [6]. Another hypothesis postulates that MICE could be induced by the procoagulant activity of invasive malignancies [3]. The present case supports a reactive origin related to prior cardiac instrumentation or chronic inflammation. Irrespective of its pathogenesis, the clinical significance of this lesion lies in its potential to be misdiagnosed as a primary or secondary neoplasm due to its solid growth pattern [6,7]. Benign neoplasms like myxoma and papillary fibroelastoma, and malignant neoplasms such as metastatic mesothelioma top the list of differential diagnoses of cardiac MICE [7,8]. Other differential diagnoses include thrombus and vegetations [1,9]. The differential diagnoses and their key histopathological features are summarized in Table 1.

**Table 1:** Differential diagnoses of cardiac MICE and their key histopathological and immunohistochemical features MICE: Mesothelial/monocytic incidental cardiac excrescences; PanCK: Pancytokeratin; EMA: Epithelial membrane antigen; WT1: Wilms tumor protein-1

Differential diagnoses	Histopathological features	Immunohistochemical markers
Thrombus [1,9]	Fibrin and RBCs, often layered; sometimes lined by endothelial cells	PanCK-negative; EMA-negative; calretinin-negative; WT1-negative; CD68-positive in histiocytes if present; CD31-positive in endothelial cells lining thrombus
Vegetation [1]	Composed of fibrin and platelets	PanCK-negative; EMA-negative; calretinin-negative; WT1-negative; CD68-positive in histiocytes if present; CD31-positive in endothelial cells if neovascularization
Metastatic carcinoma (especially mesothelioma) [3]	Malignant epithelial cells with atypia and invasion	PanCK-positive; EMA-positive; calretinin-positive; WT1-positive; CD68-negative; CD31-negative
Myxoma [7]	Stellate cells in a myxoid matrix; rarely, glandular elements may be seen	PanCK-negative in stellate cells and positive in glandular elements; EMA-negative; calretinin-positive; WT1-negative; CD68-negative; CD31-negative in stellate cells and positive in vessels
Papillary Fibroelastoma [8]	Avascular, papillary fronds lined by endothelium	PanCK-negative; EMA-negative; calretinin-negative; WT1-negative; CD68-negative; CD31-positive in endothelial cells
MICE [1,6,10]	Mesothelial, histiocytic, and varying numbers of inflammatory cells, all embedded in fibrin	PanCK-positive in mesothelial cells; EMA-negative; calretinin-positive in mesothelial cells; WT1-positive in mesothelial cells; CD68-positive in histiocytes; CD31-negative

A broader literature review on MICE reiterated the absence of malignant potential, and among cases with documented follow-up, no recurrences have been observed after excision [1,6]. Although typically asymptomatic and discovered incidentally, these lesions can occasionally cause refractory arrhythmias, pulmonary embolism, or cardiopulmonary failure [10]. In view of considerable overlap with other differential diagnoses, especially metastatic carcinomas, a pertinent clinical history and histopathological and immunohistochemical features are paramount for diagnosis of MICE.

## Conclusions

Mesothelial/monocytic incidental cardiac excrescences are rare, typically asymptomatic lesions that can closely mimic thrombi or neoplasms, leading to diagnostic challenges. In this case, MICE was identified incidentally in a patient with rheumatic mitral stenosis undergoing valve replacement, thereby highlighting its association with chronic inflammation and prior cardiac instrumentation. Discovery of the lesion in the left atrial appendage emphasizes the importance of thorough histopathological evaluation of excised cardiac tissues. Immunohistochemistry played a crucial role in establishing the diagnosis. This case underscores the necessity for increased awareness to prevent overtreatment or misdiagnosis and better understand the reactive nature of this enigmatic entity.
